# A Brain Controlled Command-Line Interface to Enhance the Accessibility of Severe Motor Disabled People to Personnel Computer

**DOI:** 10.3390/brainsci12070926

**Published:** 2022-07-15

**Authors:** Sofien Gannouni, Kais Belwafi, Mohammad Reshood Al-Sulmi, Meshal Dawood Al-Farhood, Omar Ali Al-Obaid, Abdullah Mohammed Al-Awadh, Hatim Aboalsamh, Abdelfettah Belghith

**Affiliations:** 1 Department of Computer Science, College of Computer and Information Sciences, King Saud University, Riyadh 11543, Saudi Arabia; gnnosf@ksu.edu.sa (S.G.); malsulmi@ksu.edu.sa (M.R.A.-S.); malf@ksu.edu.sa (M.D.A.-F.); oaalobaid@ksu.edu.sa (O.A.A.-O.); alawadh2a@gmail.com (A.M.A.-A.); hatim@ksu.edu.sa (H.A.); abelghith@ksu.edu.sa (A.B.); 2 Electrical Engineering and Computer Science department, Khalifa University, Abu Dhabi P.O. Box 127788, United Arab Emirates

**Keywords:** brain-computer interface, brain-controlled operating system, EEG signals processing, P300

## Abstract

There are many applications controlled by the brain signals to bridge the gap in the digital divide between the disabled and the non-disabled people. The deployment of novel assistive technologies using brain-computer interface (BCI) will go a long way toward achieving this lofty goal, especially after the successes demonstrated by these technologies in the daily life of people with severe disabilities. This paper contributes in this direction by proposing an integrated framework to control the operating system functionalities using Electroencephalography signals. Different signal processing algorithms were applied to remove artifacts, extract features, and classify trials. The proposed approach includes different classification algorithms dedicated to detecting the P300 responses efficiently. The predicted commands passed through a socket to the API system, permitting the control of the operating system functionalities. The proposed system outperformed those obtained by the winners of the BCI competition and reached an accuracy average of 94.5% according to the offline approach. The framework was evaluated according to the online process and achieved an excellent accuracy attaining 97% for some users but not less than 90% for others. The suggested framework enhances the information accessibility for people with severe disabilities and helps them perform their daily tasks efficiently. It permits the interaction between the user and personal computers through the brain signals without any muscular efforts.

## 1. Introduction

In the last decade, assistive technologies were developed to help people with severe disabilities accomplish their daily tasks, like operating system control. Different sources of information are applied to control the personal computer using, for example, the eye-tracking devices [1] and cost-effect brain-computer interface (BCI) [2]. The eye-trackers devices consist of infrared LEDs and one or more infrared cameras capturing the reflection of the infrared light off of the user’s eyes and an image processing algorithms applied to determine where the user is looking on a screen. The main difficulty of using such devices as an access technology is the so-called Midas touch problem because gaze direction is not always related to the focus of the attention, causing users to select a command against their will [1]. Brain-computer interface (BCI) is an alternative applied to control the personal computer by people with severe disabilities. It presents a channel between the human brain and the computer [3]. BCI measures brain activities and classifies specific patterns associated with specific tasks. The mental strategy determines the patterns users should produce to be interpretable by the BCI system. One of the mental strategies is attention, which is greatly important in learning and memorizing. It is a partly cognitive process that starts to operate when environmental stimuli are perceived [4]. Numerous methods have been developed to examine attention, such as the Test of Variables of Attention (TOVA), which has good external validity as it is widely used as a gold standard to detect attention. TOVA is a computerized performance test (CPT) with a monotonous hybrid Go/NoGo target-classification task design [5]. TOVA and CPT test-based methods are not applicable in real-time attention examination [4].

The electroencephalography (EEG)-based attention examination method ensures continuous examination in time, and continuous information can be gained about the actual mental state of the test subject. The second mental strategy requires external stimuli that can be visual, auditory, or somatosensory. Visual Evoked Potentials (VEPs) are a potential generated in the occipital region representing the EEG response to visual stimuli elicited by a series of flashes to the user’s eyes [6,7,8]. The exhibition of the eyes to an external stimulus generates an evoked potential at the brain’s cortical area, such as P300 and steady-state evoked potential (SVEP). The difference between P300 and VEP is that in P300, the user looks at a neutral position on the screen and focuses on the target stimuli, making P300 the only factor that discriminates target items from non-target items. While in VEP, the user directs the eye gaze to the target item, which influences a large part of the visual cortex, and the non-target influences a minor part. VEPs can be categorized according to waveform patterns into transient VEPs or Steady-state VEPs [9,10]. The scope of this study is the control of the BCI system using the evoked potential signals related to the P300 wave. P300 responses are positive deflections in EEG signals that happen in the brain activity 300 milliseconds after external visual stimuli occur [11]. The generated response can be detected by analyzing the brain activity in the electrodes covering the brain’s Parietal lobe. The P300 BCIs are characterized by the highest accuracy obtained during the detection of the evoked potentials, the classifier’s low computation resources, which permits a fast response and a shorter calibration that can be used if the obtained precision is not critical [12].

The frequent digital technology use, including web-based media, has a significant positive and negative impact on brain function and behavior. Various BCI apps may benefit brain health. For example, functional imaging scans show that internet-naive older adults who learn to search online significantly increase brain neural activity during simulated internet searches. However, the adverse effects of different web-based media include heightened attention-deficit symptoms, impaired emotional and social intelligence, impaired brain development, and disrupted sleep [13]. All these effects have a direct impact on the brain waves and should be taken on consideration after a while, six months, from using the BCI to control operating system functionalities. The acquired brain signals are always accompanied by artifacts related to physiological activities such as muscle or eye movements and non-physiological ones like the electrode’s placement, or the recording environment [14,15]. So, noise suppression should be applied to keep the valuable information.

BCI systems were validated according to the online and offline approaches. The EEG signals were recorded and immediately translated at the end of each recorded trial regarding the online approach. The offline approach was widely used in the literature because it used the existing benchmarks available on the Internet, such as the datasets provided by the BCI competition or PhysioNet. The two approaches are different in their implementation, and it should be noted that the performance obtained according to the offline approach decreased significantly compared to the online [16].

Many BCI-spellers were developed in the last few decades. An in-depth review of these systems was published in [3]. Our state-of-the-art evaluation showed that most BCI-spellers were created using the visual evoked potential called P300. The most important problems and challenging facing the P300-speller system [17]:

1. Crowding effect:t his problem arises when a target object is surrounded by similar objects, making determining the target difficult for the user. Inaccurate character distribution could cause such a problem.
2. Adjacency problem: this problem arises near the target, when the non-target flash and attract the user to produce the P300. It can be reduced by increasing the gap between the matrix elements and reducing the matrix size.
3. Fatigue: users feel tired when concentrating for a long time. It can be solved by increasing the communication rate for typing a good design of the visual paradigms.

Moreover, several paradigms were proposed to develop P300-spellers, including the Row/Column paradigm (RCP), the Single Character paradigm (SCP), the Checker Board paradigm (CBP), and the Region-Based paradigm (RBP). Table 1 present a comparison between the different P300 paradigms. This study adopted the RCP for P300 BCI-spellers. RC P300 speller was introduced by Farwell and Donchin, who developed a protocol whereby a subject is presented with a 6X6 character matrix. The rows and columns are randomly flashed with a predefined period and an inter-stimulus interval. Each row and column of the matrix is intensified according to a random sequence to spell a single character. The subject is asked to focus on the character he wants to spell. The P300 evoked potential appears in the EEG signal as a response to the intensification of a row or a column containing the desired character. By assessing the amplitude of the P300 response after each flash, the target cell is determined as the intersection of a row and a column that elicit the most significant P300. Extensive progress was made in developing other attractive applications, such as transmitting digits over the Internet [18], navigating the virtual world [19], virtual keyboard [20], controlling the robot via Internet [21], controlling an electric Car [22], phone keypad [23], and 2-D cursor [24]. These studies suggested a P300 BCI system allowing severely motor-disabled people to submit system commands to the operating system for execution.

The proposed method helps disabled people to use their personal computers by providing a complete control of computers through the command-line interface (CLI), which allows the user to submit many operations to the processing unit. The system includes:

- The signal amplifier devices permit the validation of the application according to the online approach.
- The signal processing system allows the analysis of the P300 signals and translates them to commands.
- The command-line interface performs the appropriate actions on the personal computer.

The rest of this paper is organized as follows. Section 2 presents the methodology and describes the EEG signal acquisition, the signal processing chain, and the different features of the application. Section 3 presents the experimental results. Section 4 discusses the obtained results, the evaluation criteria of EBCI systems, and the possible future research directions on embedded BCI systems. Finally, Section 4 provides concluding remarks.

## 2. Materials and Methods

The proposed brain-controlled O.S. command-line interface lets the user interact with his computer. The user can submit command-line statements for execution to manage the different aspects of the underlying operating system (file system, processes, system configuration, etc.). A Graphical User Interface (GUI) was implemented to enhance the user experience and the usability of the application. Moreover, the proposed application enables the user to run and benefit from the many programs installed on the user’s machine, such as the M.S. Office suite of applications. Upon starting one application, the user’s brain activity signals are translated into computer-mouse events or keyboard strikes.

### 2.1. System Architecture

**Figure 1:**
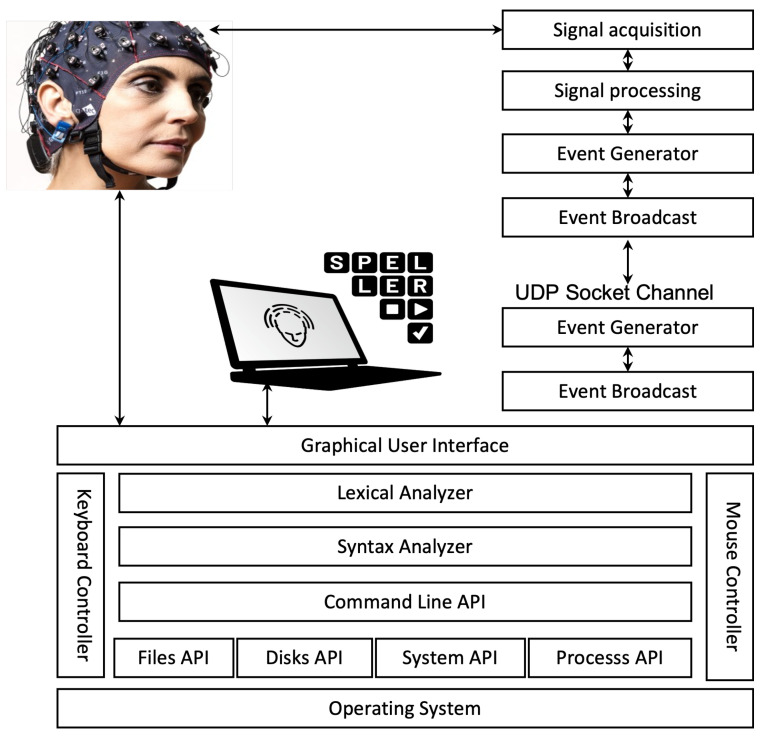
System architecture.

Figure 1 presents the general structure of the proposed system. It consists of various components that were implemented using a different programming language, such as Java and C++. The architecture comprises two subsystems: command-line interface and data acquisition system. The latter receives the brain signals from the user’s scalp in analog form and converts them to digital format to be used by the application. Moreover, the command-line interface is an editor allowing command conversion to specific services.

#### 2.1.1. Brain Controlled O.S. Command-Line Interface Architecture

The proposed O.S. Command-Line Interface architecture is composed of the following components as depicted in Figure 1:

- Graphical User Interface: It shows that the user has submitted commands, which are predicted by the P300-speller and displays the result and the status of such commands.
- Keyboard Controller: It enables users to enter text and messages using a virtual keyboard. This component is enriched by an auto-complete component that minimizes typing characters by the user.
- Mouse Controller: It enables the user to control the mouse movements by translating P300-Speller mouse commands to mouse movement services, including moving the mouse up, down, left, and right.
- Lexical Analyzer: It is responsible for parsing the commands entered by the user. It breaks the different commands entered by the user in the form of sentences into a series of tokens (lexemes). The generated tokens will be sent to the syntax analyzer.
- Syntax Analyzer: It parses a stream of tokens generated by the lexical analyzer and verifies whether the provided stream is grammatically correct or not. If so, the corresponding action of the Command-Line API is called. Otherwise, an error message is displayed.
- Command Line API: The main interface includes all the functions that the application provides, such as file system commands and O.S. commands.
- Files Library: It enables the interaction with the file system of the O.S. It allows benefiting from functionalities, such as files and directories management functions, e.g., creating, deleting, renaming, moving, and copying files.
- Disks Library: It is responsible for enabling disk driver activities provided by the system, such as Scan disks, erasing disks, organizing disks, and showing information about disk storage.
- System Library: It enables the interaction with the operating system to manage date and time, set the system configuration, and display the system history.
- Process API: It enables the interaction with the O.S. processes manager. It allows for controlling, managing, and monitoring active and background processes. It permits submitting statements to run/terminate a process, list running processes, and manage paths.

#### 2.1.2. Graphical User Interface

**Figure 2:**
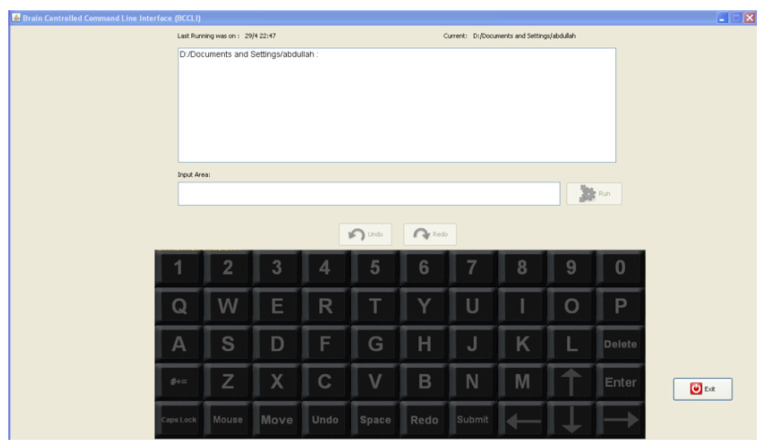
Main GUI.

Figure 2 presents a print screen of the main Graphical User Interface (GUI), which comprises three parts:

- A P300-Speller command matrix: The bottom of Figure 2 represents the different symbols/commands or characters that the user may enter. This command matrix enables the typing of character strings and activates the mouse controller.
- An input area: It shows the characters entered by the user using the P300-Speller main command/symbol matrix.
- A feedback area: This area shows the results of the commands submitted by the user and the system error messages.

#### 2.1.3. Auto-Complete Functionality

**Figure 3:**
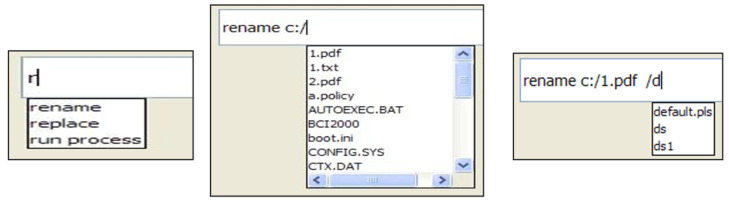
Sample of the auto-complete of a user’s command.

The auto-complete is a feature that we developed to decrease the system latency by increasing the system throughput. This feature allows the user to type a command with a minimal effort. It is linked to the input area of the GUI. Figure 3 presents a sample of the auto-complete of a user’s command. It suggests a list of available commands or file/directory or process or path names based on the few characters already entered by the user. The auto-complete feature has many advantages, such as:

- It speeds up the interaction between the user and the computer by reducing the number of characters to be typed to enter a command.
- It helps the user by suggesting a list of available commands or file/directory/process/pathnames. As shown in Figure 3, the user may rename and move a file from its location by typing a few characters.

#### 2.1.4. Moving the Mouse on the User’s Screen

**Figure 4:**
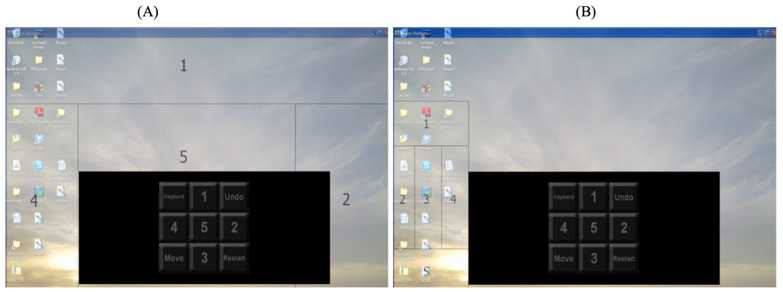
Dividing the user’s screen. (**A**) Successive division of the user’s screen into small areas. (**B**) Division the user’s screen into 5 areas.

**Figure 5:**
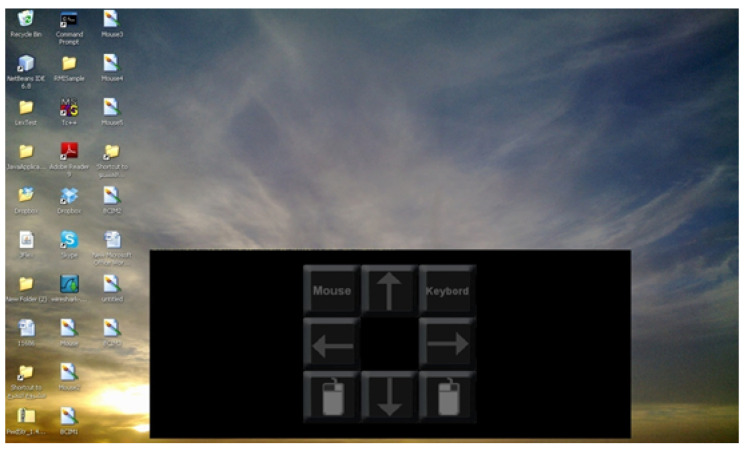
Controlling the mouse movement when no more divisions are possible.

Accessing the computer programs installed on the user’s machine, such as the M.S. Office suite of applications, was one of the main goals of the developed tool. The user’s brain activity signals will be translated into computer-mouse events, or keyboard strikes upon starting any of these applications. These features required developing a fast way to move on the user’s screen. We divided the screen into five areas, as depicted in Figure 4. A. Recursively, the division process will continue until the regions become very small. In such a case, as shown in Figure 5, a new command matrix appears, allowing the system to generate mouse events based on the desire of the user to move the mouse 5-pixels up, down, left, or right. The method may also create events corresponding to the left or right button of the mouse. Thus, the user may open files on the desktop of the computer. Moreover, this menu allows running and selecting an action from the menu of any application installed on the user’s computer.

#### 2.1.5. Structure of a Command-line Interface

Command-line Interface (CLI) is an interactive way to perform specific tasks between the user and the computer (hardware or software). A text-only interface allows typing the command and submitting it by pressing the “Enter” key. The command line usually returns the output in a text form on the same screen. The outcome may be the answer to a question or the result of an operation. Generally, CLI considers syntax and semantics at the same time. The syntax is a grammar that defines a set of rules to be respected during the call of commands. For the semantics, it establishes the order of operations to be executed. The general structure for typing a command is as follows:

- [instruction][way][destinationfiles]
- [instruction][way][destinationfiles][destinationfiles]
- [instruction][way]|[instruction][way]>[destinationfiles]

[*instruction*] means the job wants to execute it like: copy, delete, rename. [*way*] means “how to do this”. Usually, this command provides an option like delete recursively and show in sorted form. [*files*] it is the source and destination of the operations. [symbol] is a special symbol to do a specific job like (>) to move the output to a file, (|) to link two commands together to use the output of the first command as an input to the second command. A simple command line will display a command prompt which the user will write; then, the result will be in a text form or an error message based on the syntax error or permission. We notify that some CLIs allow users to change the working directory. In contrast, others protect the resources or directories and allow them only in privilege mode or with permission from the owner of these folders like kernel operation and password files.

#### 2.1.6. Class Diagram

**Figure 6:**
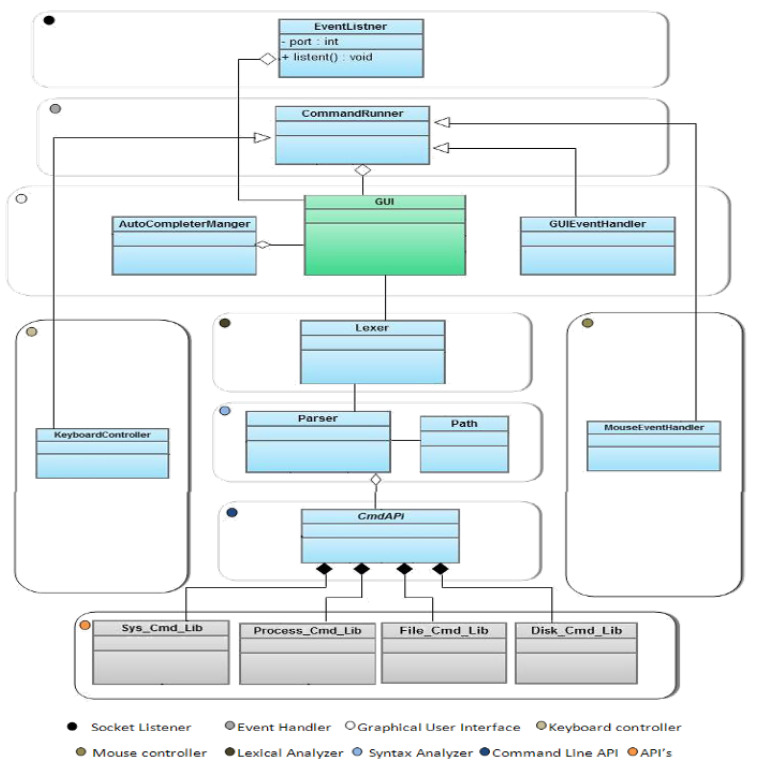
Class diagram of the proposed system.

Figure 6 presents the class diagram of the proposed application. It is composed of the following classes:

- EventListener: it is responsible for listening to a specific port to receive the command from BCI2000. Then, the command is printed at GUI.
- CommandRuner: it allows binding objects of different classes and analyzing its methods and parameters. Then, it locks up to the appropriate object and invokes the appropriate method based on what the user typed.
- GUI: it is the user interface for the system in which the inputs and outputs are printed for the user.
- AutoCompleterManger: it is responsible for listening to the text field and determining which auto-completer should activate according to what the user typed. The auto-completer feature is composed of these classes:
  1. AutoCompleter is responsible for initializing the listener and the actions listener.
  2. FileAutoCompleter extends from the AutoCompleter class. It shows the available files and directories of the file system that start with the current path typed by the user.
  3. CommandAutoCompleter extends from the AutoCompleter class. It shows the available commands that start with the current input.
- GUIEventHandler: It has functions that accept any characters and then insert them into the text area.
- MouseEventHandler: It has a function that accepts mouse events, segments the GUI to different regions, and moves the mouse cursor to this region. The GUI segmentation will continue until we reach an appropriate size.
- KeyboardController: It has an adapter that will receive keyboard commands and convert them to keyboard events for other applications such as notepad.
- Lexer: it is responsible for accepting a command and converting it to tokens.
- Parser: is responsible for analyzing the commands, which are made of a sequence of tokens, to determine their grammatical structure by respecting a given formal grammar.
- Path: it has functions that deal with the file system path. The parser class uses it.
- CmdAPI: it is an abstract layer above the APIs. It called the appropriate method of the APIs.
- Disk_Cmd_Lib: it has functions that deal with Disks operations.
- File_Cmd_Lib: it has functions that deal with the operations of files and directories.
- Process_Cmd_Lib: it has functions that deal with the process’s operations.
- Sys_Cmd_Lib: it has functions that deal with the functions of the system.

After the system startup, it shows a matrix for the user to type a command. Then, the amplifier generates signals for each character the user chooses from the matrix. These signals are handled by the BCI2000 and converted to valid commands. After that, the application receives each character from the BCI2000, and after the command is complete, it passes it to the parser to compile it and ensure it has the correct syntax. Then, according to the command, the parser is called the CmdAPI, an abstract layer of the APIs, and CmdAPI invokes the appropriate function from the APIs. Finally, the operating system performs the action, and the result will be returned and printed at GUI.

### 2.2. Signal Processing Methodology

The classification problem addressed in this study is complex and handled in two phases. The first phase is a 2-class classification problem-solving to predict if a signal, called a post-stimulus signal, corresponds to a P300 response or not. The second one deals with a multi-class classification problem since it aims to predict a symbol/command from a command matrix.

#### 2.2.1. Terminology and Annotations

Let’s consider *M* an *n* × *m* matrix of symbols (commands); *n* and *m* are the numbers of rows and columns of the matrix *M*. *M* is called a command matrix.

(1) $$M=\left[\begin{array}{ccccc} C_{11} & \cdots & C_{1j} & \cdots & C_{1m}\\ \vdots & □ & \vdots & □ & \vdots \\ \; & \cdots & \; & \cdots \\ \vdots & □ & \vdots & □ & \vdots \\ C_{n1} & \cdots & C_{nj} & \cdots & C_{nm} \end{array}\right]$$

Every row and column of the matrix *M* was assigned a unique identifier denoted rcid (row/column identifier). The following matrix shows the rcids of rows and columns of the matrix *M*. An intensification is the process of brightening (intensifying the luminosity of) all symbols of a given row or column of *M*. The previous matrix shows the intensifications of the 3rd column and of the 2nd row of a matrix of symbols respectively.

(2) $$\begin{array}{ll} & \begin{array}{ccccccc} & 1⇓ & \;□ & \;j⇓ & \;□ & \;m⇓ & \end{array}\\ \begin{array}{l} m+1\Rightarrow \\ □\\ m+i\Rightarrow \\ □\\ m+n\Rightarrow \end{array} & \left(\begin{array}{ccccc} C_{11} & \cdots & C_{1j} & \cdots & C_{1m}\\ \vdots & □ & \vdots & □ & \vdots \\ & \cdots & & \cdots & \\ \vdots & □ & \vdots & □ & \vdots \\ & \cdots & & \cdots & \end{array}\right) \end{array}$$

A sequence of intensifications *S* of the matrix *M* is an ordered collection of rows and columns of *M* intensifications. During a series of intensifications *S*, each of the rows and columns of the matrix *M* is intensified once. Hence, a sequence of intensifications *S* is composed of (n+m) distinct intensifications $<I_{1},I_{2},\ldots ,I_{n+m}>$ each of them corresponds to an intensification of a row or a column of the matrix *M*. Every intensification has two attributes:

- rank: It corresponds to the rank (first, second, third, etc.) of the intensification in a sequence *S*.
- rcid: It is the identifier of the row/column of *M* which was intensified.

**Figure 7:**
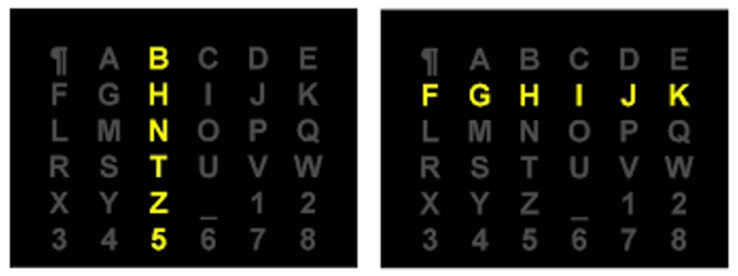
Intensifications of rows or columns of a matrix of symbols.

Figure 7 presents the order of intensifications of rows and columns of the matrix *M* during a sequence of intensifications *S*. A single sequence of intensifications *S* elicits (n+m) post-stimulus signals denoted ζ(S). ζ(S) corresponds to the following ordered collection of a post-stimulus:

(3) $$\zeta \left(S\right)=<\zeta \left(I_{1}\right),\zeta \left(I_{2}\right),\cdots ,\zeta \left(I_{n+m}\right)>\mathrm{where}\left|\zeta \left(S\right)\right|=\left(n+m\right)$$

For the selection σ of a single symbol (command) $c_{ij}$ of *M*, a sequence of intensifications *S* is repeated α times.

(4) $$\sigma =\overset{\alpha }{\underset{i=1}{⋃}}S_{i}$$

where $S_{i}$ is the *i*th repetition of the sequence *S*. Thus, the selection σ of a single command elicits a set of post-stimulus signals denoted ζ(σ):

(5) $$\zeta \left(\sigma \right)=\overset{\alpha }{\underset{i=1}{⋃}}\zeta \left(S_{i}\right)=\overset{\alpha }{\underset{i=1}{⋃}}<\zeta \left(I_{i1}\right),\zeta \left(I_{i1}\right),\cdots ,\zeta \left(I_{i(n+m)}\right)>$$

$I_{ij}$ is the *j*th intensification that occurs during the *i*th repetition of the sequence $S_{i}$ and ζ(σ) is composed of α(n+m) post-stimulus signals as depicted in the following equation:

(6) $$\left|\zeta \left(\sigma \right)\right|=\sum_{i=1}^{\alpha }\left|\zeta (S_{i})\right|=\sum_{i=1}^{\alpha }\left(n+m\right)=\alpha \left(n+m\right)$$

#### 2.2.2. Settings

Let’s consider a training data set *D* comprising post-stimulus signals corresponding to the selection of β symbols (commands) $c_{ij}$ of *M*.

(7) $$D=\overset{\beta }{\underset{i=1}{⋃}}\zeta \left(\sigma_{i}\right)=\overset{\beta }{\underset{i=1}{⋃}}\overset{\alpha }{\underset{j=1}{⋃}}\zeta \left(S_{ij}\right)$$

where $S_{ij}$ is the *j*th repetition of the sequence *S* that occurs during the *i*th selection. So, the data set *D* consists of β×α×(n+m) post-stimulus training signals.

(8) $$\left|D\right|=\sum_{i=1}^{\beta }\left|\zeta (\sigma_{i})\right|=\sum_{i=1}^{\beta }\alpha (n+m)=\beta \alpha \left(n+m\right)$$

### 2.3. Pre-Processing and Feature Extraction

The EEG signals in each electrode were filtered by an infinite impulse response (IIR) filter. It was applied to remove the unuseful information and ensure that the detected signal effectively corresponded to the P300 response. The filter block should be well tuned to avoid introducing spurious information that an inappropriate application of filter parameters could generate [15]. The applied bandpass filter was set to eight, which kept the data between 1 and 10 Hz.

### 2.4. Classification Strategy

In the case of a single classifier, all the training data set *D* signals were used to train a 2-class classifier. The classifier was trained to predict if a signal contained a P300 response or not. Given a post-stimulus signal corresponding to an intensification *I* of a row/column of *M*, the prediction method, denoted ρ, returned a value of 1 or 0, which indicated whether the signal ζ(I) was a P300 response or not.

(9) ρ(ζ(I))=vsuchthatv∈{0,1}

The evoked potentials arise about 300 ms after the stimulus; this time window is enough to capture all required time features for efficient classification. The extracted signals are filtered with an 8th order bandpass filter to keep the bins between 0.1 and 10 Hz. The filtered signals have been decimated according to 10 Hz, which results in a signal composed of 14 samples. Finally, a post-stimulus signal has been transformed into a vector by concatenating the 14 samples of all the 64 channels. So, for each subject, the training portion comprises 15,300 post-stimulus vectors of dimension 896, for which labels are v∈{0,1}.

Given a sequence of intensifications *S*, the parsing method denoted τ returned a row vector with values obtained by using the prediction method ρ.

(10) τ(ζ(S))=U

*U* is a row vector composed of the elements $<u_{1},u_{2},\ldots ,u_{n+m}>$ such that $u_{i}=$$\rho (\zeta \left(I_{\left(j\in [1\cdots n+m\right]}\right)))$ where $I_{j}.rcid=i$. As such,

(11) τ(ζ(S))=<ρ(ζ(I.rcid=1)),ρ(ζ(I.rcid=2)),⋯,ρ(ζ(I.rcid=n+m))>

The parsing method τ identified which intensifications of rows/columns of *M* were elicited, during the sequence *S*, P300 responses and which were not.

Given a selection σ, the α sequences of intensifications were parsed sequentially by the single classifier using the parsing method τ leading to α row vectors, each of which corresponded to $\tau {\left(\zeta \left(S_{i}\right)\right)}_{1\leq i\leq \alpha }$. The α row vectors $\tau {\left(\zeta \left(S_{i}\right)\right)}_{1\leq i\leq \alpha }$ were then used to calculate the probability that the intensifications of rows and columns of *M* that occurred during the selection σ elicited P300 responses. These probabilities were computed using the following prediction function:

(12) $$\begin{array}{c} \Psi \left(\zeta \left(\sigma \right)\right)=<u_{1},\cdots ,u_{m},u_{m+1},\cdots ,u_{m+n}>\;\\ \mathrm{such}\;\mathrm{that}u_{i}=\frac{1}{\alpha }<\sum_{i=1}^{\alpha }\rho (\zeta \left(I_{j}.rcid=i\right)) \end{array}$$

where $I_{j}.rcid=i$ is the intensification of the row/column of *M*, in which rcid was equal to *i*, that was performed within the *j*th sequence $S_{j}$ during the selection σ.

(13) $$\begin{array}{c} \Psi \left(\zeta \left(\sigma \right)\right)=<\frac{1}{\alpha }<\sum_{i=1}^{\alpha }\rho (\zeta \left(I_{j}.rcid=i\right)),\cdots ,\;\\ \frac{1}{\alpha }<\sum_{i=1}^{\alpha }\rho (\zeta \left(I_{j}.rcid=n+m\right))> \end{array}$$

Let *y* be the rcid of the *M* column that had most probably elicited P300 responses. *y* is the identifier of the column that maximizes the score $u_{y}$.

(14) $$u_{y}=Max_{i=1}^{m}\left(u_{i}\right)$$

Let *x* be the rcid of the *M* row that has most probably elicited P300 responses. *x* is the identifier of the row that maximizes the score $u_{m+x}$.

(15) $$u_{x}=Max_{i=m+1}^{\left(m+n\right)}\left(u_{i}\right)$$

We consider that the symbol $c_{xy}$ of *M* is the most probably user’s desired symbol.

## 3. Results

The proposed system was validated according to the online approach for comparison and benchmarking purposes and regarding the online approach to check the stability of the system in term of accuracy with real users.

### 3.1. Offline Testing

The offline testing was performed using a public dataset provided by the BCI competition [25]. It contained the EEG signals recorded from two different subjects and five spelling sessions. According to the offline approach, the system was validated using a public dataset containing P300 post-stimulus signals recorded from two subjects during five spelling sessions. For each subject, the training set is composed of 85 characters spelling. Every character spelling corresponds to 180 = 12 × 15 post-stimulus labeled signals (each of them collected over 64 channels). Thus, for each subject, the training dataset corresponds to 15,300 = 85 × 180 post-stimulus labeled signals. In the meantime, for each subject, the testing set is composed of 100 characters spelling which correspond to 18,000 = 100 × 180 post-stimulus signals. Table 2 sums up the different parameters of the dataset using the previous mathematical modeling.

### 3.2. Online Testing

**Figure 8:**
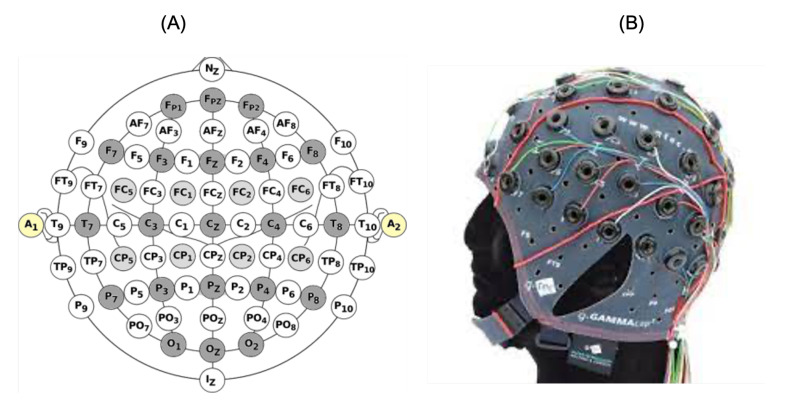
EEG electrode localization. (**A**) electrode positions. (**B**) g. GAMAcap.

The online testing was performed using the acquisition system G.Nautilus Research, which allowed the non-invasive recording of the brain activities from the scalp through 64 dry electrodes. The EEG signals were captured and digitalized using a highly accurate analogic to digital converter with 25-bit accuracy at a 250 Hz sampling rate. The electrodes were placed. The amplifier was connected to a P.C. using a USB cable, and the management was performed using a C Application Programming Interface (C API). The electrodes were placed on the scalp according to the international position system 10–20 as depicted in Figure 8A. The proposed application was controlled through the P300 signals, and the related brain activities mainly appeared in the cortex area. Therefore, only the electrodes in this region were kept during the acquisition (Fz, Cz, Pz, P4, P3, Oz, P08, P07) and placed on the scalp via the g.GAMAcap as illustrated in Figure 8B. The skin preparation needed low impedance between the skin and electrodes, so a conductive gel was used. The management of the G.Nautilus Research amplifier was assured by an open-source framework called BCI2000. This framework was implemented using the C++ language. BCI2000 comprises four modules to handle brain signals: the source module to acquire signals, the processing module to treat EEG signals, the application module to take the user feedback, and the operator module. Furthermore, the BCI2000 configures the P300 speller window, such as the size and location of the window, spelling modes, spelling matrix, and destination address used to submit the user’s selections to the user’s application.

## 4. Discussion

The proposed framework allows users with severe disabilities to control most of the functionalities of an operating system, mainly by brain activities. It helps them submit commands to the terminal, enter text and messages, control mouse movements, interact with the files systems, manage the process, etc. The signal processing algorithm was tested according to the online and offline approaches and used different classification algorithms for comparison purposes to select the suitable one with maximum performance in terms of accuracy [26]. In this respect, we implemented four classifiers from different families: Linear Discriminant Analysis (LDA) [14], Partial Least Squares regression (PLS) [27], logistic regression (REG) [11], and Support Vector Machine (SVM) [28].

The system performance was evaluated based on its accuracy, defined by the ratio between the correctly classified trials and the total number of investigated trials. Table 3 shows the accuracy of the system obtained by the different classifiers. The SVM method outperformed the other algorithms for P300 detection, providing an accuracy average of 94.5%. In contrast, the accuracy of the SVM classifier reached 96% for subject A where the LDA, PLS, and REG algorithms achieved 93%, 94%, and 94%, respectively. However, the SVM failed to keep the highest accuracy for subject B, where the PLS and REG algorithms outperformed the SVM and the LDA.

For comparison purposes, Table 4 summarizes the accuracy obtained by the adopted classification strategy and those obtained by the winners of the BCI competition. The proposed classification strategy with the simple feature extraction and the filtering technique outperformed the 2nd and 3rd ranked algorithms. Compared to the winner, the proposed system reached the same accuracy of 96% for subject A, and the accuracy decreased by 3% only for subject B.

The system was validated according to the online approach by connecting an acquisition system to the computer and asking five participants to use the different APIs of the proposed environment. Furthermore, the subjects were requested to minimize any source of physiological artifacts that could deteriorate the system performance, such as avoiding eye movement or any muscular activities during the use of the system. The obtained accuracies were very promising and tended to be 97% for some subjects, and in the least, the obtained accuracy was about 90%. The obtained accuracies exceeded the requirement of the navigation accuracy by 20% [16]. The promising accuracies obtained according to the online approach clearly showed the efficiency of the proposed method as the accuracy was maintained above 70% for all subjects. The experimental results encouraged the use of the proposed system by people with severe disabilities as it guaranteed a good performance in terms of accuracy.

In [29], a file explorer based on BCI mouse is proposed allowing the user to access a computer and manipulate the stored files. The framework integrates the common function of an explorer such as BCI mouse, BCI speller, and an explorer. BCI mouse is controlled by the P300 and motor imagery signals to move the cursor from an arbitrary initial position to an arbitrary target position, and further select a target of interest or reject an unintended target. The BCI speller is implemented based on P300 speller to enter the path. The explorer is composed from the BCI mouse and the P300 speller allowing to perform window’s explorer basic function (e.g., access a fold in the computer, open, close, copy, paste, and delete a file in this fold). Shenghong et al. proposed an asynchronous hybrid BCI system integrating a speller, web browser, an email-client and a file explorer controlled by the EEG and electrooculography (EOG) signals [30]. The file explorer API is validated on ten healthy subjects and the average accuracy reached 95% and the and the time spent performing a selection/rejection is about 1.76 min using the motor imagery signals against 1 min using the P300 signals [29]. The GUI integrates few features only such as the movement of the cursor and the selection of icons. Mathias presented in [31] a simple GUI allowing user to control mouse cursor and keyboard input on the level of operating system, thereby making it possible to use any windows application such as notepad. The system reached an average accuracy of 85% in a free-spelling mode, which enable participant to write 21 error-free characters per minute. Compared to the previous systems, the proposed framework contains more features and cover the most functionalities of an operating system instead on focus on a simple feature as the launch of a software. Furthermore, it contains a command line interface, lexical and syntax analyzer, file and disks libraries management, and process management. In term of the accuracy, the platform outperformed than the framework presented in [31] where the accuracy is enhanced by a 10%.

## 5. Conclusions

This study presents an integrated computational environment using brain-computer interface (BCI) technology to enhance the accessibility to information and open a new opportunity for assisting people with severe disabilities in their daily life activities. Using the extended and customized open-source software tools, our proposed environment provides a new opportunity to interact with graphical user interfaces via brain activities to manage computer resources. The proposed system enables a complete control of the personal computer with the minimum number of commands, giving the user more comfort and satisfaction. The signal processing chain is based on an IIR filter to remove artifacts and different classifiers to predict which symbol/command of a command matrix is the user’s target command. The average accuracy of the proposed system achieved 94.5% using the SVM classifier.

In the future, we intend to extend the proposed system to include the control of the developed APIs to be used by subjects with eyesight problems. Finally, we want to expand the framework to have a web browser, which edits API to write documents and gives the user a wider control and a more independent environment.

## Figures and Tables

**Table 1:** P300 paradigms Comparison.

	RCP	SCP	CBP	RBP
Accuracy	Medium	Low	Very high	High
Adjacency problem	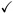	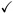	×	×
Crowding Effect	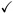	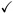	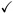	×
Double flash	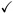	×	×	×

**Table 2:** Description of the benchmarking dataset.

Parameter	Notation	Formula	Value
The number of rows of the command Matrix *M*.	*n*		6
The number of columns of the command Matrix *M*.	*m*		6
The number of signals during a single sequence *S*.	|ζ(S)|	|ζ(S)|=n+m	12
The number of sequences *S* in a single selection σ.	α		15
The number of post-stimulus signals during a single selection.	|ζ(σ)|	|ζ(S)|=α(n+m)	180
The number of selections (per subject) of the training dataset.	β		85
The number of post-stimulus signals (per subject) of the training dataset.	|D|	|D|=βα(n+m)	15,300
The number of selections (per subject) of the testing dataset.	$\beta^{\prime }$		100
The number of post-stimulus signals (per subject) of the testing dataset.	$|D^{\prime }|$	$|D^{\prime }|=\beta^{\prime }\alpha \left(n+m\right)$	18,000

**Table 3:** Asymmetrical pairs between happy and pleased emotions.

Subject	LDA	SVM	PLS	REG
Subject A	93	96	94	94
Subject B	92	93	94	94

**Table 4:** Accuracy obtained by the different classifiers (%).

		Winner of BCI Competition		
**Subject**	**Proposed Method**	**1st**	**2nd**	**3rd**
Subject A	96	96	90.5	80
Subject B	93	95	90.5	80

## Data Availability

Not applicable.
